# Acute Peritoneal Dialysis in a Patient with Severe Uremic Syndrome and Multiple Hemodialysis Access Failure

**DOI:** 10.1155/2024/8891887

**Published:** 2024-08-05

**Authors:** Made Dyah Vismita Indramila Duarsa, Gede Wira Mahadita, Yenny Kandarini

**Affiliations:** Rumah Sakit Umum Pusat Sanglah Denpasar, Denpasar, Indonesia

## Abstract

A 67-year-old woman was diagnosed with chronic kidney disease stage V, severe uremia syndrome, hyperkalemia, metabolic acidosis, suspected pulmonary oedema, and multiple hemodialysis access failure. The patient is in a condition that requires emergency hemodialysis, but the patient does not have any access to undergo hemodialysis. The patient then underwent acute peritoneal dialysis and received an adequate response. The patient continued continuous ambulatory peritoneal dialysis and responded well.

## 1. Introduction

Chronic kidney disease (CKD) is a progressive chronic disease that has high morbidity and mortality rates. CKD can be classified into five stages based on GFR levels. CKD stage V is a stage that requires kidney replacement therapy [1]. Hemodialysis is one of the kidney replacement therapy options widely used in CKD patients, but hemodialysis cannot be done without vascular access. When all vascular access cannot be used, the patient is declared to have multiple hemodialysis access failures [2].

Uremic syndrome patients with multiple hemodialysis access failures are life-threatening; other renal replacement therapy options must be considered when hemodialysis cannot be performed. Acute peritoneal dialysis is one of the renal replacement therapy options that can be used. Until now, acute peritoneal dialysis has been rarely performed, only around 11% of all dialysis cases globally [3].

A rare case of a 67-year-old woman undergoing acute peritoneal dialysis because the patient experienced acute uremic syndrome with multiple hemodialysis access failures is reported in this case report.

## 2. Case Presentation

A 67-year-old female presented for routine dialysis; however, the hemodialysis access became obstructed throughout the procedure. The patient complained of increasingly severe shortness of breath. Based on examinations, the patient was diagnosed with CKD stage V, suspected pulmonary oedema, hyperkalemia, metabolic acidosis, and type 2 diabetes mellitus. The patient's current condition is an indication of emergency dialysis. Installing access to the right and left femoral vein failed due to a suspected thrombus. The left and right internal jugular veins also cannot be used due to previous tunnelling of the double-lumen catheter. The patient was diagnosed with multiple hemodialysis access failure, meaning hemodialysis could not be carried out because the patient did not have any access. The patient has had a history of CKD stage V for the last four years and has undergone routine hemodialysis. The patient also has a history of type 2 diabetes mellitus for more than 20 years. The patient denied any history of other diseases.

**Figure 1:**
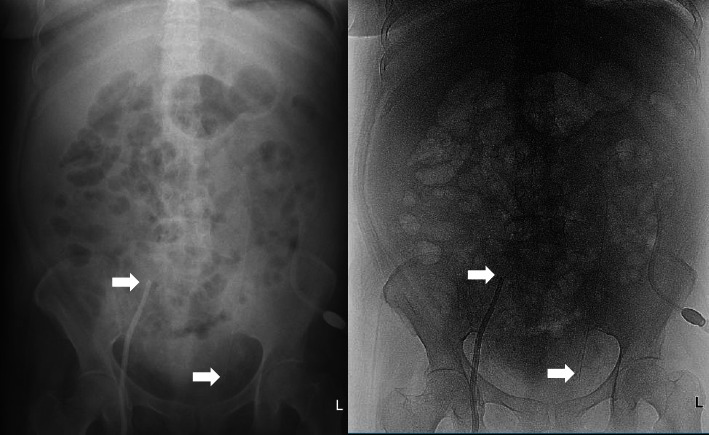
Plain photo of the patient's abdomen. Arrows: Dower catheter and Tenckhoff catheter tips.

At a team meeting, it was decided that the patient would undergo acute PD as a possible renal replacement therapy. The first acute PD was performed immediately, 1 hour after the Tenckhoff catheter was inserted (Figure 1). Trained PD nurses initiated acute PD in the supine position. The first and second PD processes are described according to Tables 1 and 2. The patient immediately provides an adequate response after the PD process. Complaints of tachypnea and shortness of breath decreased. Evaluation of laboratory examinations after serial PD also obtained improved results, according to Table 3. The frequency of dwelling and the amount of dialysis increased gradually during the hospital stay. Starting with 1 liter every 2 hours using 1.5% and 2.5% dialysate fluid alternately on the first day up to 1.5 liters every 6 hours using three times 1.5% dialysate fluid and at night with dialysate fluid 2.5%. The fluid balance in and out of the dwelling process slowly produces the expected results (Table 4).

After eight days of treatment, the patient was allowed to be discharged from the hospital. The patient is in stable condition without complaints. Two months after outpatient, the patient had no symptoms and received an adequate response. The patient continued Continuous Ambulatory Peritoneal Dialysis and responded well.

## 3. Discussion

The uniqueness of this case report was the use of acute PD as a renal replacement therapy in a patient with severe uremic syndrome. Initially, a double-lumen catheter (DLC) insertion procedure was planned to tunnel the old DLC at the right femoral vein. This is aimed to providing access to emergency hemodialysis due to the patient's worsening condition. During the surgery, an attempt to overwire the old CDL of the right femoral vein failed due to a right iliac thrombus. Attempts on the contralateral side also failed due to a thrombus. The left and right internal jugular veins were unusable because they were already damaged due to previous DLC tunnelling. The patient also has malfunctioning vascular access on the right and left brachiocephalic arteriovenous fistula. Consequently, due to multiple hemodialysis access failures, conventional hemodialysis could not be done [4, 5].

While literature suggests using a temporary central venous catheter (CVC) for emergency dialysis, it is typically removed and transitioned to PD once the patient stabilizes to limit exposure to CVC-related complications [6, 7]. A Cochrane review indicates that acute PD has a lower risk of bacteremia than CVC for initiating hemodialysis [8]. Despite limited research comparing PD with CVC, a study by Onime et al. reported successful PD in a patient with multiple failed vascular accesses, improving the patient's quality of life over three years [9]. In a retrospective study of 30 patients with hemodialysis access failure, 20 were transferred to PD, 7 continued with hemodialysis after vascular access placement in nonstandard settings, and 3 patients underwent renal transplantation [10]. Mortality rates were highest in the nonstandard hemodialysis group (85%) and lowest in the PD group (15%). These findings suggest that PD is superior to CVC for emergency dialysis in acute settings [10].

Another alternative treatment for this condition is kidney transplantation, but kidney transplantation is not possible in this case due to the absence of a donor. Also, the option of kidney transplant in acute conditions is still controversial. The arteriovenous graft was also not viable for this emergency case because it still needs time for use [6, 8, 11–13]. So, PD becomes the only suitable alternative for renal replacement therapy in this case.

PD is a dialysis method that inserts dialysate into the peritoneal cavity through a catheter using the peritoneal membrane as a semipermeable filter [14, 15]. Acute PD can begin within two weeks of catheter insertion. Several researchers have suggested the term “urgent-start PD” for patients who require dialysis in less than 72 hours, and there is also the term “emergency-start PD” for patients who require dialysis immediately after the Tenkchoff catheter placement [6, 16, 17]. In practice, most acute PD patients begin within 4-5 days after the catheter placement. In this case, PD was carried out only 1 hour after catheter placement.

Although limited studies comparing acute dialysis therapy options, several studies have shown no significant difference between acute PD, conventional PD, or emergency hemodialysis in clinical outcomes or prognosis in patients [6, 8, 11–13]. Acute PD was chosen in this patient with the initial goal of improving metabolic conditions. Short dwelling time and high frequency in the acute phase are aimed to increase the effectivity of toxic elimination and removal of excess fluid [18]. This approach aims to reduce complications such as dialysis leakage or peritonitis and facilitate the body's adaptation to PD. PD focuses on removing small and medium clearance molecules and a few large ones [19]. Later, when the patient has begun to stabilize, we will adjust with a longer dwelling and higher fluid concentration.

According to the literature, severe hyperkalemia or pulmonary oedema is a relative contraindication of acute PD [20]. Acute PD is often avoided because hemodialysis is considered more effective in this condition [6, 16]. However, given the inability to perform hemodialysis and the lack of alternative therapies, acute PD was pursued despite the patient's pulmonary oedema and hyperkalemia.

This study did not evaluate primary diseases, such as diabetes mellitus and low serum albumin levels, as a factor predicting survival or complications. Actually, this was not necessary because the patient was in an emergency condition to get dialysis (hyperkalemia, metabolic acidosis, shortness of breath, and suspected pulmonary oedema), and the patient had no options other than peritoneal dialysis at this time. In addition, the DM in this case was controlled. Indeed, in conventional PD, diabetes mellitus and low serum albumin levels are becoming predicting factors for complications and poor prognosis that need to be considered before the initiation of conventional PD [21, 22]. Studies have shown that PD patients with DM and or low serum albumin levels have a higher risk of mortality than patients without DM. This is attributed to the increase in infection risk, peritonitis, cardiovascular complications, and endothelial dysfunction in diabetes mellitus [21, 22].

Icodextrin was not used in the acute setting due to the need for short dwelling times and high frequency. However, it can be considered for long-term use once the patient stabilizes, as it maintains a stable osmotic gradient and reduces glucose absorption risk compared to conventional glucose dialysate, making it safer for diabetic patients. Hence, it is safer for DM patients because it does not cause a significant increase in blood sugar levels [23].

This study has several limitations. First, the patient presented with multiple failed vascular accesses, and initial blood vessel assessments were not conducted by our hospital. Second, this study did not report the result of peritoneal equilibration tests (PET) as PET can only be performed 3-4 weeks postinitiation. Acute PD offers significant benefits for acute dialysis, including maintaining residual renal function, preserving vascular access sites, and reducing costs compared to emergency hemodialysis. Additionally, it is a viable option in areas without hemodialysis facilities [14, 15]. Despite catheter-related complications appear to be higher in acute PD than conventional PD, survival and clinical outcomes are comparable [6, 8, 13]. Thus, we suggest considering acute PD as a renal replacement therapy option for patients with multiple vascular access failures that require acute dialysis.

## 4. Conclusion

This case report shows that acute peritoneal dialysis is a safe and feasible option in patients with indications for acute dialysis. There were no complaints in patients with severe uremic syndrome after the acute peritoneal dialysis. This action provides good clinical outcomes for patients.

## Figures and Tables

**Table 1:** 1^st^ peritoneal dialysis.

Input time	Inflow	Output time	Outflow	Balance	Total dwelling time	RR	HR	Symptoms
16.30	500	17.30	450	+50	1 hour	20	116	(−)
18.05	500	19.05	100	+450	2 hours	20	110	(−)
19.55	500	20.55	500	+450	3 hours	20	109	(−)
21.21	1000	22.21	700	+750	4 hours	20	107	Abdominal discomfort, shortness of breath (−)
22.48	1000	23.48	700	+1050	5 hours	20	108	Abdominal discomfort, constipation
00.00	300	09.00	1100	+250	19 hours	20	113	(−)

**Table 2:** 2^nd^ peritoneal dialysis.

Input time	%	Inflow	Output time	Outflow	Balance	Total *dwelling* time	RR	HR	Symptoms
10.00	1.5	1000	12.30	1100	−100	2.5 hours	21	113	Abdominal discomfort
12.30	2.5	1000	14.30	1200	−300	4.5 hours	23	114	(−)
14.30	1.5	1000	16.30	700	0	6.5 hours	20	113	(−)
16.45	2,5	1000	18.45	1850	−850	8.5 hours	20	100	(−)

**Table 3:** Evaluation of patient laboratory examination results.

Parameter	Pre-PD	Post 1^st^ PD	Predischarge from hospital	Unit
Urea	91.9	84.4	73.1	mg/dL
Creatinine	20.7	19.6	17.9	mg/dL
eGFR	1.5	1.6	1.79	
pH	7.25	7.24	7.36	
pCO_2_	35	39	41	mmHg
HCO_3_	15.3	16.70	23.2	mmol/L
BEecf	−11.9	−10.7	−2.2	mmol/L
Na	134	137	136	mmol/L
K	6.5	5.2	4.2	mmol/L

**Table 4:** Patient's peritoneal dialysis course during treatment.

Day	Inflow	Outflow	*Ultrafiltration/*fluid balance	Total *dwelling* time (jam)
1	3800	3550	+250	19
2	4000	4850	−850	8.5
3	5000	5500	−500	10
4	5000	5800	−800	10
5	6000	6900	−900	10
6	7000	8300	−1300	12.5
7	7500	8300	−800	13
8	7500	8300	−800	15
				

## References

[1] Kalantar-Zadeh K., Jafar T. H., Nitsch D., Neuen B. L., Perkovic V. (2021). Chronic kidney disease. *The Lancet*.

[2] Brown R. S. (2020). Barriers to optimal vascular access for hemodialysis. *Seminars in Dialysis*.

[3] Bello A. K., Okpechi I. G., Osman M. A. (2022). Epidemiology of peritoneal dialysis outcomes. *Nature Reviews Nephrology*.

[4] Johnson R. J., Feehally J., Floege J. T. (2019). *Comprehensive Clinical Nephrology*.

[5] Muaddi L., Ledgerwood C., Sheridan R., Dumont T., Nashar K. (2022). Acute renal failure and its complications, indications for emergent dialysis, and dialysis modalities. *Critical Care Nursing Quarterly*.

[6] Rastogi A., Lerma E. V., Bargman J. M. (2021). Applied Peritoneal Dialysis: Improving Patient Outcomes. Applied Peritoneal Dialysis: Improving Patient Outcomes.

[7] Ghaffari A. (2012). Urgent-start peritoneal dialysis: a quality improvement report. *American Journal of Kidney Diseases*.

[8] Htay H., Johnson D. W., Craig J. C., Teixeira-Pinto A., Hawley C. M., Cho Y. (2021). Urgent-start peritoneal dialysis versus haemodialysis for people with chronic kidney disease. *The Cochrane Database of Systematic Reviews*.

[9] Onime A., Tzamaloukas A. H., Servilla K. S., Hartshorne M. F. (2007). Peritoneal dialysis as salvage renal replacement therapy after complete failure of hemodialysis access in an elderly patient with multiple comorbidities. *Advances in Peritoneal Dialysis*.

[10] Gameiro J., Fonseca J. A., Jorge S., Lopes J. A. (2018). Management of end-stage vascular access failure patients: a retrospective analysis. *Portuguese Journal of Nephrology and Hypertension*.

[11] Parapiboon W., Sangsuk J., Nopsopon T. (2022). Randomized study of urgent-start peritoneal dialysis versus urgent-start temporary hemodialysis in patients transitioning to kidney failure. *Kidney International Reports*.

[12] Niang A., Iyengar A., Luyckx V. A. (2018). Hemodialysis versus peritoneal dialysis in resource-limited settings. *Current Opinion in Nephrology and Hypertension*.

[13] Tadayon N., Abedi A. R. (2023). Unveiling a pseudoaneurysm at the anastomosis site in a transplanted kidney: an initial misclassification as hydro nephrosis—a case report and literature reviewA case report and literature review. *Urology Case Reports*.

[14] Kandarini Y. (2022). *Continuous Ambulatory Peritoneal Dialysis. I Gde Raka Widiana*.

[15] Gde Raka Widiana I., Kandarini Y., Paramita Ayu N., Ketut Suardana N. S. (2022). *Terapi Dialisis Buku Pegangan untuk Dokter dan Perawat Dialisis*.

[16] Yaxley J., Scott T. (2023). Urgent-start peritoneal dialysis. *Nefrología*.

[17] Krediet R. T., Furgeson S., Teitelbaum I. (2023). The Physiology and Pathophysiology of Peritoneal Transport. Nolph and Gokal’s Textbook of Peritoneal Dialysis.

[18] Borrelli S., La Milia V., De Nicola L. (2019). Sodium removal by peritoneal dialysis: a systematic review and meta-analysis. *Journal of Nephrol*.

[19] Li Y., Pi H. C., Yang Z. K., Dong J. (2020). Associations between small and middle molecules clearance and the change of cognitive function in peritoneal dialysis. *Journal of Nephrol*.

[20] Ponce D., Zamoner W., Dias D. B., Banin V., Balbi A. L. (2023). Advances in peritoneal dialysis in acute kidney injury. *Revista de Investigacion Clinica; Organo del Hospital de Enfermedades de la Nutricion*.

[21] Meng L. F., Yang L. M., Zhu X. Y. (2020). Comparison of clinical features and outcomes in peritoneal dialysis-associated peritonitis patients with and without diabetes: a multicenter retrospective cohort study. *World Journal of Diabetes*.

[22] Shi Y., Cai J., Shi C., Liu C., Zhou J., Li Z. (2022). Low serum albumin is associated with poor prognosis in patients receiving peritoneal dialysis treatment. *Journal of Healthcare Engineering*.

[23] Frampton J. E., Plosker G. L. (2003). Icodextrin: a review of its use in peritoneal dialysis. *Drugs*.

